# Linking vitamin B1 with cancer cell metabolism

**DOI:** 10.1186/2049-3002-1-16

**Published:** 2013-07-24

**Authors:** Jason A Zastre, Rebecca L Sweet, Bradley S Hanberry, Star Ye

**Affiliations:** 1 Department of Pharmaceutical and Biomedical Sciences, College of Pharmacy, University of Georgia, R.C. Wilson Pharmacy Building, Athens, GA 30602, USA

**Keywords:** Thiamine, Transketolase, Vitamin, Metabolism, Cancer

## Abstract

The resurgence of interest in cancer metabolism has linked alterations in the regulation and exploitation of metabolic pathways with an anabolic phenotype that increases biomass production for the replication of new daughter cells. To support the increase in the metabolic rate of cancer cells, a coordinated increase in the supply of nutrients, such as glucose and micronutrients functioning as enzyme cofactors is required. The majority of co-enzymes are water-soluble vitamins such as niacin, folic acid, pantothenic acid, pyridoxine, biotin, riboflavin and thiamine (Vitamin B1). Continuous dietary intake of these micronutrients is essential for maintaining normal health. How cancer cells adaptively regulate cellular homeostasis of cofactors and how they can regulate expression and function of metabolic enzymes in cancer is underappreciated. Exploitation of cofactor-dependent metabolic pathways with the advent of anti-folates highlights the potential vulnerabilities and importance of vitamins in cancer biology. Vitamin supplementation products are easily accessible and patients often perceive them as safe and beneficial without full knowledge of their effects. Thus, understanding the significance of enzyme cofactors in cancer cell metabolism will provide for important dietary strategies and new molecular targets to reduce disease progression. Recent studies have demonstrated the significance of thiamine-dependent enzymes in cancer cell metabolism. Therefore, this review discusses the current knowledge in the alterations in thiamine availability, homeostasis, and exploitation of thiamine-dependent pathways by cancer cells.

## Review

### Vitamin B1

**Figure 1:**
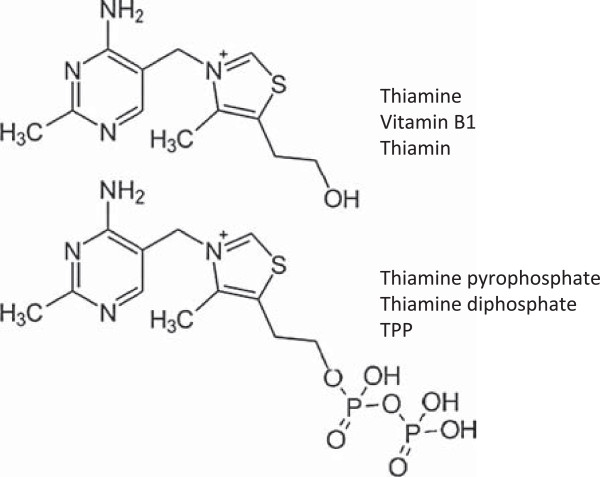
Chemical structures of thiamine (vitamin B1; thiamin) and the active co-enzyme thiamine pyrophosphate (thiamine diphosphate; TPP).

Thiamine is classified as an essential water-soluble vitamin requiring continuous dietary intake to support carbohydrate metabolism. Thiamine is critical for the activity of four key enzymes in cellular metabolism, pyruvate dehydrogenase (PDH) and alpha-ketoglutarate dehydrogenase (α**-**KGDH) in the tricarboxylic acid (TCA) cycle, transketolase (TKT) within the pentose phosphate pathway (PPP), and branched chain alpha-keto acid dehydrogenase complex (BCKDC) involved in amino acid catabolism. Structurally, thiamine is composed of a thiazole and pyrimidine ring joined together by a methylene bridge (Figure 1). Although thiamine is not the co-enzyme but is converted to the active diphosphate, thiamine pyrophosphate (TPP) form intracellularly, circulating plasma levels of thiamine in healthy individuals range between 10 and 20 nM [1]. The recommended daily intake (RDI) of thiamine for adult men and women is approximately 1–1.5 mg/day [2]. Thiamine is found naturally in many foods including breads, fish, meat, eggs, legumes and milk, as well as being used in fortification of many processed foods (Table 1). In addition, many over-the-counter vitamin supplements contain a significantly large quantity of thiamine representing 100 to 6,600% of the RDI (Table 1).

**Table 1:** Amount and percent daily value (DV) of thiamine found in food and supplements

**Dietary source**	**Thiamine (mg)**	% DV^**^
Natural^*^		
Pork, fresh (3 oz)	0.6	40
Fish (1/2 fillet)	0.3	20
Black beans (1 cup)	0.4	27
Lima beans (1 cup)	0.3	20
Potatoes (1 cup)	0.3	20
Okra (1 cup)	0.2	13
Chicken (1 cup)	0.2	13
Peas (1 cup)	0.2	13
Sunflower seeds (1 cup)	0.7	47
Pistachios (1 oz)	0.2	13
Pecans	0.2	13
Fortified^*^		
General Mills, total raisin bran (1 cup)	1.6	107
General Mills, total corn flakes (1.3 cups)	1.5	100
Breadcrumbs (1 cup)	1.2	80
White rice (1 cup)	1.1	73
Submarine sandwich, with cold cuts (6" sandwich)	1.0	67
Cornmeal (1 cup)	0.9	60
**Supplements**		
Centrum		
Adult	1.5	100
Child (≥4 yrs)	1.5	100
One A Day		
Women’s 50+	4.5	300
Women	1.5	100
Girl Teen	2.3	153
Men’s 50+	4.5	300
Men	1.2	80
Solaray		
Boy Teen	3.8	250
B Complex	7.5	500
Nature’s Way		
Vitamin B1	100	6,667
Nature Made		
Vegan B Complex	25	1,667
B Complex	15	1,000

^*^Values obtained from United States Department of Agriculture (USDA).

^**^The % DV is the percentage of the recommended daily intake (RDI) that each food/supplement contains. The RDI (1–1.5 mg) is determined based on the estimated average requirement of thiamine in that only a small percentage (approximately 2 to 3%) of individuals may experience deficiency [2].

### Vitamin B1 homeostasis

**Figure 2:**
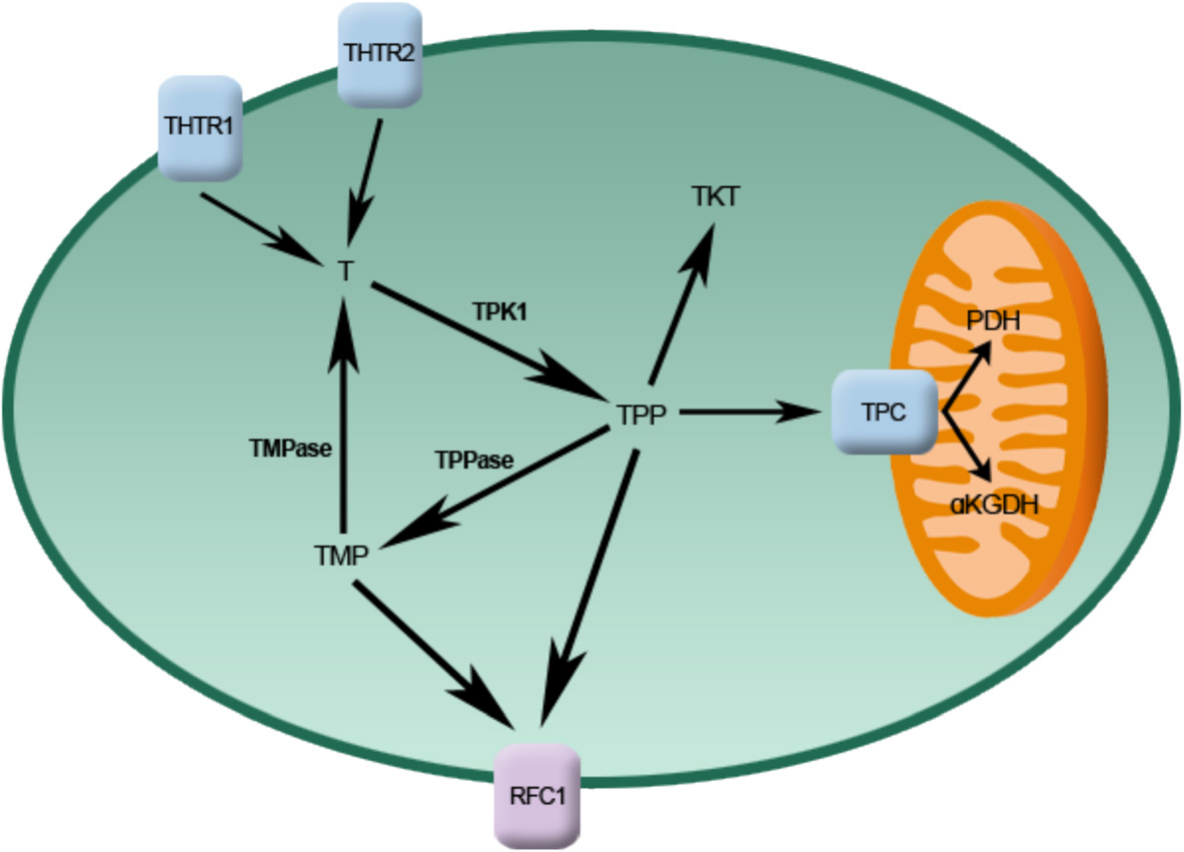
**Intracellular thiamine homeostasis is initially achieved through the uptake of thiamine (T) by the thiamine transporters THTR1 and THTR2.** Once inside the cell, thiamine is converted to the active co-enzyme, thiamine pyrophosphate (TPP) by thiamine pyrophosphokinase-1 (TPK1). Thiamine can than function as a cofactor for the cytoplasmic TKT. Transport of TPP by the thiamine pyrophosphate carrier (TPC) across the mitochondrial membrane supplies cofactor for activity of pyruvate dehydrogenase (PDH) and alpha-ketoglutarate dehydrogenase (α-KGDH). Intracellular TPP can also be converted to thiamine monophosphate (TMP) by thiamine pyrophosphatase (TPPase) and subsequently recycled back to thiamine by thiamine monophosphatase (TMPase). Both TMP and TPP can be effluxed out of the cell through the reduced folate carrier (RFC1).

The quaternary nitrogen and overall hydrophilicity of thiamine necessitates a carrier-mediated process for absorption and cellular uptake (Figure 2). Two transporters belonging to the *SLC19A* family, THTR1 (*SLC19A2*) and THTR2 (*SLC19A3*) primarily facilitate the transport of thiamine. The other member of the *SLC19A* family, RFC1 (*SLC19A1*) facilitates intracellular uptake of reduced folate [3]. Although all three transporters share a high degree of amino acid sequence similarity, RFC1 does not transport thiamine and THTR1/2 has not been shown to transport reduced folate or other organic cations [4,5]. *SLC19A2* and *SLC19A3* transport thiamine with K*m* values of 2.5 μM and 27 nM, respectively [6,7]. Intestinal absorption of thiamine has also been described to occur by passive diffusion mechanisms at high concentrations and also via members of the organic cation transporter family [8-10]. Recently, a high affinity carrier-mediated transport mechanism for TPP has been characterized in intestinal cells but no transporter has been identified [11].

Upon transport into the cell, thiamine is converted to the active co-enzyme thiamine pyrophosphate (diphosphate) by thiamine pyrophosphokinase-1 (TPK1) (Figure 1) [12]. Phosphorylation of thiamine by TPK1 has been shown to be a significant driving force for thiamine uptake along with binding to apo-enzymes [13]. Human TPK1 exists as a homodimer and is expressed ubiquitously with high levels in the kidney, small intestine, and testis [12,14]. In addition to TPP, three other phosphorylated forms have been observed intracellularly in humans, thiamine monphosphate (TMP), thiamine triphosphate (TTP), and adenosine thiamine triphosphate (AThTP) [1]. Although the physiological functions of TMP, TTP, and AThTP have not been ascertained, TPP is the only known thiamine phosphorylate functioning as an enzyme cofactor. Dephosphorylation of intracellular TPP by thiamine pyrophosphatase to TMP can be subsequently recycled back to free thiamine via thiamine monophosphatase [15,16]. *SLC19A1* has been shown to efflux the mono and diphosphate ester of thiamine [17]. It is unclear if the dephosphorylation and efflux of thiamine phosphorylates is to regulate intracellular thiamine levels to control cofactor and non-cofactor functions of thiamine phosphorylates. Transport of TPP across the mitochondrial membrane to support PDH and α-KGDH activity is facilitated by the thiamine pyrophosphate carrier (TPC), which is encoded by the *SLC25A19* gene [18]. Previously identified as a deoxynucleotide carrier or DNC, homology comparisons to yeast TPP transporter and transport assays have clearly identified TPC as a TPP transporter [18,19].

### Thiamine homeostasis genes and cancer

A difference in the expression of thiamine homeostasis genes in cancer has been extensively demonstrated for the thiamine transporter *SLC19A3*. Using a cDNA array, Liu *et al*. demonstrated a decrease in *SLC19A3* expression in breast cancer compared to corresponding normal tissue [20]. Down-regulation of *SLC19A3* was also found in gastric and colon cancer [21,22]. The decrease in expression appears to involve epigenetic repression through hypermethylation and histone deacetylation of the *SLC19A3* promoter [21,22]. An elevation of *SLC19A3* methylation was detected in the plasma of early- and advanced-stage breast cancer patients as well as gastric cancer [23]. Recently, our group discovered a significant increase in the gene expression of TPK1, *SLC19A2*, and *SLC25A19* in breast cancer tissue samples compared to normal breast tissue [24]. A slight decrease in *SLC19A3* gene expression was also found in concordance with previous findings. Upregulation of TPK-1, *SLC19A2*, *SLC25A19* and downregulation of *SLC19A3* was also verified in several breast cancer cell lines compared to human mammary epithelial cells (hMECs). Although *SLC19A3* expression was repressed, transport assays demonstrated an increase uptake of thiamine in all breast cancer cell lines tested compared to hMECs. Using HPLC to quantify thiamine and thiamine phosphorylates, the overall thiamine status (total of all thiamine forms assayed) was significantly greater in two of the four cancer cell lines evaluated compared to hMECs. Interestingly, all four breast cancer cell lines exhibited a high level of intracellular free thiamine compared to TPP. This may suggest an important non-cofactor role of thiamine in cancer cells or a mechanism to retain the intracellular thiamine supply to support binding to newly synthesized apo-enzyme in progenitor or newly formed daughter cells.

Why tumor cells repress the expression of THTR2 is still unclear. Decreasing expression of one thiamine transporter may not impact overall thiamine status, since transport may still occur via THTR1. This may indicate additional functionality of THTR2 beyond facilitating the intracellular transport of thiamine. When exogenously re-expressing *SLC19A3* in gastric cancer cells, a marked decrease in growth rate was found [21]. Liu *et al*. demonstrated that THTR2 transfection in breast cancer cells increased sensitivity to ionizing radiation and cytotoxicity to doxorubicin [21]. However, it is unclear if the growth-suppressive properties observed in these two studies are mediated through an increase in cellular thiamine concentrations from enhanced transport capacity or due to a novel pro-apoptotic function of THTR2. To this end, Liu *et al*. established the changes in gene expression when overexpressing THTR2 and when grown in thiamine-deficient media [25]. Several genes involved in oncogenesis were upregulated with THTR2 overexpression that was reduced in the absence of exogenous thiamine [25]. The increase in oncogenic genes with THTR2 re-expression would appear to contradict a role for THTR2 as a tumor suppressor but highlights a potential role for thiamine and THTR2 expression in tumorigenesis.

Though the expression of SLC19A3 appears to be repressed in a number of cancers, Sweet *et al*. demonstrated that hypoxic exposure resulted in upregulation or re-establishment of *SLC19A3* expression in breast cancer cells [26]. A corresponding 2-fold increase in thiamine transport was also observed, suggestive of an increased requirement for thiamine during hypoxic stress [26]. The presence of hypoxic microenvironments in tumors results in an increase in glycolytic activity, primarily controlled by the hypoxia-inducible factor-1 alpha (HIF-1α) transcription factor. Knockdown of HIF-1α attenuated the upregulation, suggesting that *SLC19A3* may be an associated gene involved in the adaptive metabolic shift during hypoxic stress [26]. Interestingly, thiamine was shown to reduce hypoxia-mediated apoptosis of rat cardiomyocytes [27]. Thus, increasing transport activity and intracellular thiamine supply may be part of a pro-survival response during hypoxic stress. In this capacity, it is unclear whether thiamine is functioning as a cofactor or non-cofactor. Evidence suggests that the mitochondrial injury associated with hypoxia can lead to an imbalance of reactive oxygen species (ROS) in cancer cells [28-30]. Unchecked, the excess ROS can lead to cellular apoptosis and necrosis and has been exploited as a chemotherapeutic approach [31,32]. Thiamine has direct antioxidant properties as well as being essential for glutathione production [33-35]. Therefore, clarification of thiamine’s role in pro-survival responses to hypoxic stress would be of great significance, given the association of hypoxia with poor patient prognosis.

### Thiamine and cancer

It has been hypothesized that a Western diet, characterized in part by excess thiamine supplementation, may be a factor for increased cancer incidence compared to other countries [36]. Thiamine is commonly supplemented in processed foods and readily consumed in over-the-counter vitamin and nutritional supplements in Western countries with generally high cancer incidences. In contrast, Asian and African countries principally consume food that is high in thiaminase, a natural thiamine-degrading enzyme, which may reduce thiamine exposure [36]. Although no direct studies have evaluated this hypothesis, several have attempted to correlate the intake of thiamine and other nutritional components with the risk of cancer. However, like so many other nutritional correlations with cancer incidence, the dietary intake of thiamine and cancer risk has provided conflicting results. Using nutritional questionnaires and a calculated average daily intake level, patients with prostate cancer consume less thiamine than those without cancer suggesting a negative association with cancer risk [37]. A 2008 study examined the relationship between the intake of B vitamins and incidence of breast, endometrial, ovarian, colorectal, and lung cancer in women [38]. No correlation was found between intake of the B vitamins, including thiamine, riboflavin, niacin, and folate, and the incidence of cancer. Interestingly, reduced thiamine intake increased the number of aberrant crypt foci in the colons of rats fed a sucrose-based diet [39]. Patients with severe malnutrition have exhibited Baker’s cyst, osteosarcoma, and submandibular gland cysts that were cured without recurrence after thiamine administration, suggesting a role of thiamine deficiency in tumor development [40].

A limited number of studies and case reports have determined the overall thiamine status in cancer patients. Clinically, thiamine status is quantified biochemically using a TKT assay of whole blood samples [41]. This assay involves measuring the increase in the activity of the thiamine-dependent enzyme TKT after added TPP. If deficient in thiamine, exogenous TPP will stimulate TKT activity, termed the TPP effect. Basu *et al*. demonstrated that patients with advanced cancer exhibit a greater TPP stimulating effect, suggestive of a reduced thiamine status [42,43]. Similarly, increased TPP effect was characterized in patients with B-chronic lymphocytic leukemia, Burkett’s lymphoma, and acute myelomonocytic leukemia [44,45]. Using an HPLC assay to directly quantify TPP levels in whole blood, Tsao *et al*. demonstrated a significant decrease of TPP in patients with advanced stages of non-small cell lung cancer [46]. Although the reason for a decrease in thiamine status in the blood is unclear, one study noted that cancer patients had a higher level of thiamine urinary excretion [42]. The authors suggested that the decrease in thiamine status might not be due to reduced dietary intake of thiamine, but an inability to activate thiamine to TPP [42]. However, thiamine status is primarily assayed biochemically in whole blood and limited studies have quantified thiamine/TPP directly in cancer tissue. The reductions in peripheral thiamine/TPP may be a consequence of extensive accumulation and/or utilization by cancer cells. Trebukhina *et al*. demonstrated that tumor growth resulted in a depletion of tissue vitamin stores and an increase in the TPP-stimulating effect in blood [47]. During tumor growth, cancer cells maintained a constant level of TPP while host liver tissue exhibited a perpetual decline [48]. In post-surgical or autopsy tissues, a 2.5-fold increase in thiamine levels was found in colon adenocarcinomas relative to un-invaded control tissue [49]. Overall these studies strongly suggest a preferential accumulation of thiamine into cancer cells that may be responsible for the alteration in peripheral thiamine status during malignancy.

Aside from the disease itself, chemotherapeutic drugs such as 5-fluorouracil (5-FU) and ifosfamide have been associated with inducing a thiamine-deficient state in patients [50,51]. In most cases, patients exhibit neurological impairment similar to the sequelae observed in thiamine deficiency conditions such as Wernicke’s encephalopathy [51-54]. How these drugs that are structurally unrelated to thiamine are capable of inducing a deficiency is unknown and may involve distinct mechanisms. No change in thiamine or TPP levels was found in patients receiving ifosfamide treatment, suggesting that ifosfamide or a metabolite may inhibit a thiamine-dependent pathway [51,55]. Accumulation of thiamine was found to be increased in cancer cells and rat hepatocytes when treated with 5-FU and doxifluridine [56]. The enhancement in thiamine uptake by 5-FU in cancer cells has been associated with an increase in intracellular TPP while free thiamine remained constant [56]. Treatment of rats for three consecutive days with 5-FU was found to result in an increase in the TPP-stimulating effect on whole blood TKT, and a decrease in liver thiamine stores [57]. Thus, 5-FU appears to decrease peripheral thiamine levels by increasing cellular accumulation and conversion to TPP. Although the mechanism for either drug still needs continued research, high-dose thiamine is capable of reversing the symptoms of thiamine deficiency [50,51,53,54].

Self-supplementation vitamin preparations containing levels of thiamine greater than the RDI are readily accessible and considered to be safe and harmless for patients (Table 1). Although the use of thiamine to treat deficiency-related symptoms attributed to the disease or therapy is warranted, this is currently done with limited comprehension of the role thiamine may contribute towards malignant progression. In light of our knowledge regarding alterations of thiamine homeostasis in cancer, the impact of thiamine supplementation on cancer growth has received minimal research attention. In 2001, Comin-Anduix *et al*. evaluated the effect of increasing thiamine supplementation in multiples of the RDI on an Ehrlich ascites tumor-mouse model [58]. Their findings indicated a statistically significant stimulatory effect of thiamine supplementation on tumor growth compared to non-supplemented controls. Moderate doses of 12.5 to 37.5 times the RDI had the greatest stimulatory effect, peaking at approximately 250% greater tumor cell proliferation with 25 times the RDI. Interestingly, at values above 75 times the RDI, no change was found in tumor cell proliferation, and a slight decrease was found at 2,500 times the RDI. This observation suggests that there is a specific range in which thiamine supports proliferation. A recent study explored the relationship between a high-fat diet and thiamine levels on the tumor latency in the Tg(MMTVneu) spontaneous breast cancer-tumor mouse model [59]. In this study a normal-fat (NF) diet contained 10% of the calories from fat while the high-fat diet contained 60%. Low thiamine (LT) levels were defined as 2 mg of thiamine per 4,057 kcal and normal thiamine (NT) levels as 6 mg per 4,057 kcal. Tumor latency was significantly longer (295 days) in animals given a NF/LT diet compared with animals on NF/NT (225 days). Interestingly,the delay in tumor latency from LT was abolished when given a high-fat diet. This demonstrates an important interplay of dietary constituents on tumor progression that needs further characterization. Although more research is needed to confirm and evaluate the role of thiamine on disease progression, these studies have significant clinical implications. First, patients requiring thiamine to treat either chemotherapy or disease-associated deficiency should receive high-dose thiamine to avoid enhancing tumor growth. Second, self-supplementation of thiamine by cancer patients should be avoided as the low-to-moderate levels of thiamine may contribute to disease exacerbation.

The importance of thiamine in cancer cell proliferation is highlighted by studies using the thiamine-degrading enzyme thiaminase. Liu *et al*. demonstrated that the addition of thiaminase into cell culture media containing thiamine had a significant growth inhibitory effect on breast cancer cells [60]. Thiaminase reduced ATP levels in cancer cells, demonstrating thiamine’s key role in support of cancer cell bioenergetics. Moreover, a pegylated version of thiaminase was capable of delaying tumor growth and prolonging survival in an RS4 leukemia xenograft model [61]. Thiamine’s key role in cancer cell metabolism and survival is further demonstrated by studies using the thiamine analog oxythiamine, which functions as an anti-coenzyme and is capable of reducing *in vivo* and *in vitro* tumor cell growth [62-64]. Inhibition of TKT by oxythiamine reduces DNA and RNA synthesis through reductions in ribose 5-phosphate (R5P) synthesis, the pentose carbon backbone of all nucleotides. Oxythiamine also has been shown to induce apoptosis in rat PC-12 cells via mitochondria-dependent caspase 3-mediated signaling pathways [65]. The effect on nucleotide synthesis is highlighted by the prominent G1 cell cycle arrest induced by oxythiamine in Ehrlich’s tumor cells [63]. Yang *et al*. demonstrated that oxythiamine decreases cell migration and invasion *in vitro*, as well as reduced lung metastases in mice with Lewis lung carcinoma (LLC) [66]. Interestingly, oxythiamine did not reduce the proliferation of LLC cells at concentrations that reduced migration and invasion. The effect of oxythiamine was attributed to a dose-dependent reduction in MMP-2 and MMP-9 activity and expression. This finding suggests that thiamine-dependent pathways have other repercussions on cancer progression in addition to effects on cellular proliferation.

### Thiamine-dependent enzymes in cancer

#### Transketolase

**Figure 3:**
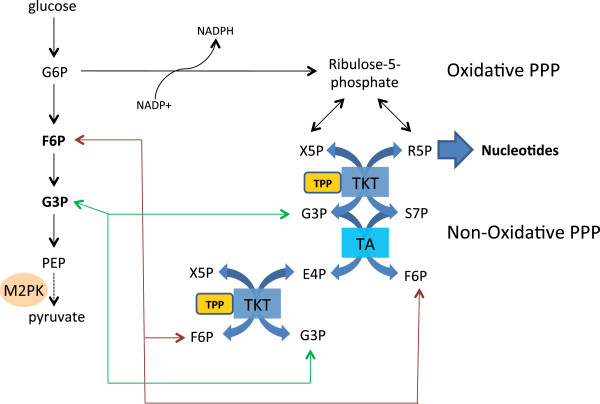
**Under normal cell metabolism G6P entering the oxidative pentose phosphate pathway (PPP) is converted to ribose 5-phosphate (R5P) and xylulose 5-phsophate (X5P).** Both can be further metabolized through the non-oxidative pathway to ultimately form fructose 6-phosphate (F6P) and glyceraldehyde 3-phosphate (G3P) that re-enters the glycolytic pathway to continue catabolism for ATP production. In cancer, reduced activity of M2-PK leads to an excess of F6P and G3P that can be shunted back into the non-oxidative pathway for anabolism. Mediated through transaldolase (TA) and the TPP-dependent enzyme TKT, F6P and G3P are converted to R5P for biosynthesis of nucleotides.

The PPP and in particular the thiamine-dependent enzyme TKT is essential for cancer cells to synthesize large amounts of nucleic acids needed for rapid cellular growth (Figure 3). In normal cells, glucose 6-phosphate enters the PPP and is converted to the nucleotide pentose sugar R5P through the non-thiamine-dependent oxidative branch. If not utilized for *de novo* nucleotide synthesis, R5P continues into the non-oxidative branch of the PPP where TKT ultimately converts R5P into fructose 6-phosphate (F6P) and glyceraldehyde 3-phosphate (G3P), which re-enters the Embden-Meyerhoff pathway. In contrast, a shift to increased reliance on the non-oxidative PPP for R5P production is found in cancer cells. Boros *et al*. demonstrated that 98% of the ribose molecules in H441 lung cancer cells were derived through the non-oxidative pathway [67]. Similarly, 85% of the ribose RNA in pancreatic adenocarcinoma cells was from the non-oxidative pathway [62].

One of the key regulators of glycolysis in cancer cells is the M2 isoform of pyruvate kinase (M2-PK), which catalyzes the conversion of phosphoenolpyruvate to pyruvate. M2-PK is highly expressed in rapidly proliferating cells and is the predominant isoform in cancer [68]. Depending on the metabolic needs, M2-PK oscillates between the active tetramer and inactive dimeric form [69]. The dimer, also known as tumor M2-PK, is the predominant form of PK found in cancer cells and is a potential biomarker for cancer detection [70-73]. The reduced PK activity induces a build-up of phospho-metabolites such as F6P and G3P that are utilized by TKT to produce R5P [74]. Recently, metabolic profiling of gliomas was consistent with an anabolic signature exhibiting reduced M2-PK activity, a build-up of glycolytic intermediates, and high R5P production [75]. Resistance of chronic myeloid leukemia cells to imatinib is associated with an increase in M2-PK expression and an increase in glucose flux into RNA through the non-oxidative PPP [76,77]. Thus, the alteration in glycolysis regulation redirects glucose carbon into thiamine-dependent anabolic pathways that support rapid proliferation and cell survival.

Although the TKT reaction is generally considered to be the result of a single TKT gene, there are two additional TKT isoforms found in the human genome, termed TKT-like 1 (TKTL1) and TKT-like 2 (TKTL2). The increase in substrate flux through the non-oxidative PPP may be supported by an overexpression of TKT isoforms. Immunohistochemical staining of malignant tissues, including breast, lung, colonic, urothelial, ovarian, endometrial, gastric and laryngeal tissues, have all exhibited an overexpression of TKTL1 compared to normal tissues [78-86]. High expression of TKTL1 is correlated with tumor progression and poor patient prognosis [78,79,85,87]. TKTL1 silencing has been shown to suppress growth and proliferation within various tumor cell lines and xenograft models [88-93]. Ectopically overexpressing TKTL1 in head and neck squamous cell carcinoma cells were found to increase cellular proliferation over vector control [94]. Cells overexpressing TKTL1 consumed more glucose, produce greater amounts of G3P, F6P, pyruvate, lactate, and R5P, indicating that TKTL1 promotes an aerobic glycolysis phenotype. Overexpression of TKTL1 has also been associated with activation of the pro-oncogenic HIF-1α under normoxic conditions [94]. The PPP and TKTL1 activity may be important in pro-survival responses by limiting excess production of ROS when challenged with oxidative stresses, such as hypoxia. The glycolytic flux through the oxidative PPP generates nicotinamide adenine dinucleotide phosphate (NADPH), which is essential in regenerating glutathione and for biosynthetic reactions. Increased oxidative stress in cancer cells was demonstrated to suppress M2-PK activity and enhance flux through the oxidative PPP with a concomitant decrease in reduced glutathione levels [95]. Interestingly, knockdown of TKTL1 expression was found to reduce cellular NADPH and glutathione levels with a reciprocal increase in ROS-mediated apoptosis [91]. When exposed to hypoxic stress, reducing TKTL1 expression has resulted in an increase in ROS generation and cell death to glioma cells [96]. It is unclear how TKTL1 activity in the non-oxidative PPP is related to NADPH production generated within the oxidative pathway. TKTL1 activity may contribute to NADPH formation by maintaining continuous flux of glucose carbons through the PPP to avoid intermediate feedback on enzymatic activity.

Although the importance of TKTL1 in cancer proliferation, survival, and metabolism is well established, several reports have suggested that TKTL1 is incapable of enzymatically functioning as a TKT. Homology comparisons of TKTL1 with TKT have noted a deletion of 38 amino acids within the cofactor and catalytic domain, suggesting that TKTL1 is incapable of binding to TPP and carrying out the TKT reaction [97,98]. Schneider *et al*. engineered a 38 amino acid deletion pseudo-TKTL1 (TKTΔ38) mutant from TKT as a model of TKTL1 to elucidate this issue [99]. No conventional TKT activity of the TKTΔ38 mutant was detectable using a coupled spectrophotometric assay for the conversion of known physiological substrates. Moreover, circular dichroism and proton nuclear magnetic resonance (1H-NMR) spectroscopy indicated that the TKTΔ38 mutant had no associated TPP. Using the same TKTΔ38 mutant system, Meshalkina *et al*. confirmed the lack of TKT activity in solution and were unable to detect TPP after acid or heat denaturation extraction methods [100]. However, the lack of activity of purified TKTΔ38 in solution contradicts reports describing the TKT activity of TKTL1 when exogenously overexpressed or repressed in cancer cells [76,91,93,94]. Since TKT functions as a homodimer, it is unclear if the TKTΔ38 mutation alters dimer formation. Even if TKTL1 is enzymatically inactive as a homodimer, the expression of TKTL1 in mammalian systems may influence overall TKT activity through heterodimer formation with other TKT isoforms. The lack of TPP binding to the TKTΔ38 mutant does not preclude other thiamine derivatives from binding. The diphosphate group added to thiamine does not participate in the catalytic activity and functions primarily to anchor the cofactor into the apo-enzyme. Other thiamine derivatives with unknown function have been found intracellularly; these may be able to bind within the condensed catalytic site of TKTL1 [1]. Thus, further work is needed to fully understand the biochemistry of TKTL1 in mammalian systems.

How thiamine supplementation impacts carbon flux through the non-oxidative pathway and modulates TKT activity in cancer cells is unknown. Increases in TKT activity may be attributed to an upregulation of enzyme expression. Additionally, thiamine supplementation may also stimulate TKT activity by maintaining a high holo-enzyme fraction by binding to apo-enzymes in progenitor and/or newly generated daughter cells. Recent work describing the benefit of thiamine supplementation in reducing hyperglycemia-induced vascular damage in diabetes may provide some insight. Similar to cancer, hyperglycemia can result in a build-up of phosphometabolites in non-insulin-dependent tissues such as the vascular endothelia. This diverts glucose metabolites into the polyol, hexosamine, advanced glycation, and the diacylglycerol pathways, which are associated with inducing hyperglycemic vascular damage [101]. High-dose thiamine, or the thiamine derivative benfotiamine, stimulate TKT activity and decrease production of toxic metabolites [102-104]. The exposure of high-dose thiamine to human umbilical vein endothelial cells and bovine retinal pericytes cultured in high glucose has been found to result in an increase in TKT mRNA expression and TKT activity [105]. Conversely, thiamine deficiency was found to decrease TKT mRNA levels and reduce TKT activity in neuroblastoma cells [106]. Thus, thiamine appears to have both a regulatory and stimulatory effect on TKT activity.

#### Pyruvate dehydrogenase

**Figure 4:**
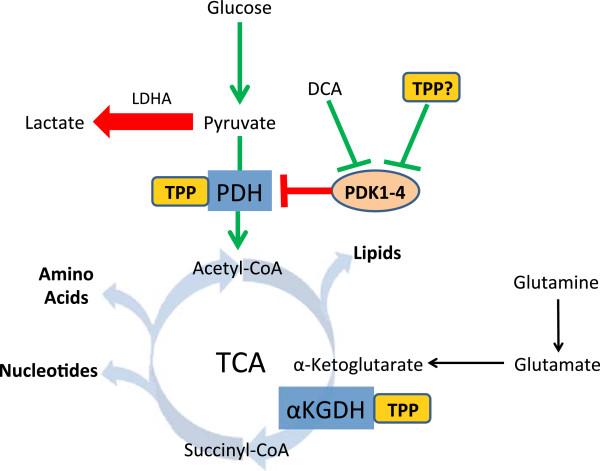
**The pyruvate dehydrogenase (PDH) complex converts pyruvate into acetyl-CoA when bound to the co-enzyme, thiamine pyrophosphate (TPP).** In cancer, phosphorylation of PDH by pyruvate dehydrogenase kinase isoforms 1 to 4 (PDK1 to 4) inactivates PDH, leading to a reduction, in pyruvate conversion to lactate by lactate dehydrogenase A (LDHA). Inhibition of PDK activity by dichloroacetate (DCA) reduces phosphorylation and induces apoptosis. TPP binding to PDH has also been suggested to inhibit PDK phosphorylation and may explain why high dose thiamine has anti-proliferative effects in a tumor xenograft model. In the oxidative direction, pyruvate is converted to acetyl-CoA by PDH and is continually catabolized to α**-**ketoglutarate. The thiamine-dependent enzyme alpha-ketoglutarate dehydrogenase (α**-**KGDH) converts α**-**ketoglutarate to succinyl-CoA. Cancer cells exploit glutaminolysis to resupply the tricarboxylic acid (TCA) cycle with carbon as α**-**ketoglutarate. Continuation of the TCA cycle results in anabolic activity to provide precursors for nucleotides, amino acids, and lipids for biomass generation.

The conversion of pyruvate to acetyl-CoA takes place through a series of reactions mediated by the thiamine-dependent enzyme PDH. Located within the mitochondrial matrix, PDH is a multi-component enzyme complex consisting of three subunits (E1, E2, and E3). As a result of its location at the junction between glycolysis and the TCA cycle, PDH activity functions as a critical gatekeeper for the continued metabolism of glucose (Figure 4). The activity of PDH is tightly regulated through phosphorylation by PDH kinase (PDK) [107]. PDK is a family of four isoenzymes (PDK1, 2, 3 and 4) that function to inhibit PDH activity through ATP-dependent phosphorylation [108-110].

In normal cells PDH is active, allowing cells to maintain oxidative metabolism to produce ATP and other components necessary for cell survival and proliferation. However, PDH activity is suppressed in cancer due to downregulation and overexpression of PDK isoforms [111-114]. This truncation of glucose metabolism, highlighted by preferential conversion of pyruvate to lactate, provides cancer cells with a metabolic advantage to maintain rapid proliferation [115]. Thus, an increase in thiamine availability to cancer cells would not be expected to further increase carbon flux through PDH. However, PDH expression is elevated with a reciprocal decrease in PDK isoform expression in tumor-associated stromal tissue, such as fibroblasts and vascular endothelial cells [114,116]. The increased PDH activity in surrounding tissues has been proposed to assist in the detoxification of extracellular lactate produced by cancer cells [116]. This metabolic symbiosis between cancer cells and stromal tissue may be enhanced through increasing thiamine availability, promoting PDH activity in normal tissue surrounding tumors.

Interestingly, restoration of PDH activity in cancer cells has been shown to promote apoptosis and is actively being assessed as a potential therapeutic strategy. One such compound, DCA, has shown considerable promise in multiple cancer types to inhibit PDK-mediated phosphorylation of PDH [117,118]. The re-establishment of glucose oxidation in cancer cells through restored PDH activity results in an increase in mitochondrial ROS and cytochrome C release, ultimately leading to apoptosis [119,120]. Regulation of PDK activity is mediated through the accumulation of metabolic products such as ATP, nicotinamide adenine dinucleotide (NADH) and acetyl-CoA, which stimulate activity, while pyruvate and ADP inhibit when in excess [121]. Another regulator of PDH phosphorylation is the thiamine cofactor TPP, which, when bound to PDH, reduces the rate and extent of PDK-mediated phosphorylation [110]. Thus increasing concentrations of TPP through thiamine supplementation may be pro-apoptotic through restoration of PDH activity in cancer cells. This may explain why a reduction in tumor growth was observed with high-dose thiamine supplementation [58]. The potential of high-dose vitamin B1 to reduce cancer cell growth would be of particular significance and warrants further study.

#### Alpha-ketoglutarate dehydrogenase

In addition to glucose, cancer cells extensively undergo glutaminolysis, utilizing glutamine as a carbon and nitrogen source [122,123]. Upon entering the cell, glutamine is deaminated to form glutamate and ultimately α-ketoglutarate (α-KG), which serves as an anaplerotic substrate to replenish the TCA cycle (Figure 4). Located in the mitochondria, α-KGDH mediates the conversion of α-KG to succinyl-CoA. With α-KGDH being located at a critical junction that connects glutaminolysis with the TCA cycle, understanding the impact of thiamine supplementation on the functional contribution of α-KGDH to cancer metabolism is highly desirable. Unfortunately there are no reports to date describing changes in α-KGDH expression or activity in cancer.

In the oxidative direction, glutamine derived α-KG entering the TCA cycle is a key carbon backbone for the synthesis of amino acids and nucleotides [122]. Alternatively, glutamine-derived α-KG can undergo reductive decarboxylation through the TCA cycle, forming citrate for lipid biosynthesis [124,125]. This reverse TCA carbon flow would bypass the requirement for α-KGDH activity, allowing the cell to utilize α-KG unabated by changes in thiamine availability. It is unclear how α-KGDH activity is regulated to allow α-KG to move in the reverse direction but has been described to be triggered during hypoxic stress [125,126]. Normally, the activity of α-KGDH is stimulated by low concentrations of calcium and ADP and inhibited at high NADH and succinyl-CoA levels [127]. Reduced activity of α-KGDH has been observed during high oxidative stress that may provide a regulatory switch that allows for reverse TCA carbon flow during hypoxia [128]. Several studies have evaluated the effects of thiamine deficiency on expression and activity of α-KGDH with conflicting results. In human lymphoblasts, fibroblasts and neuroblastoma cells, no change in α-KGDH gene expression and activity were observed during thiamine deficiency [106]. In contrast, *in vivo* studies reported decreased α-KGDH activities in the neuronal tissues during thiamine deficiency [129,130]. Therefore, clarification of α-KGDH regulation and what impact changes in thiamine availability have on activity and directional flux of the TCA cycle is greatly needed in cancer.

#### Branched chain alpha-keto acid dehydrogenase complex

Valine, isoleucine, and leucine are essential branched chain amino acids (BCAAs) that can serve as an energy source as well as precursors for amino acid and protein synthesis [131,132]. The metabolism of BCAAs involves transamination to the α**-**keto acid followed by irreversible oxidative decarboxylation by the thiamine-dependent BCKDC to form an acyl-CoA derivative [132]. The continued catabolic breakdown of BCAA produces acetyl-CoA (from leucine) and succinyl-CoA (from valine and isoleucine) that enter the TCA cycle [132,133]. BCKDC is a multi-component enzyme consisting of three subunits (E1, E2, and E3) and is located in the mitochondria [132]. Similar to PDH, BCKDC activity is regulated through reversible phosphorylation by branched-chain α**-**keto acid dehydrogenase kinase (BDK) and phosphatase (BDP) [134]. Loss-of-function mutations and BCKDC deficiencies are associated with accumulation of neurotoxic α**-**keto acids, referred to as maple syrup urine disease (MSUD) that is characterized by a sweet urine odor [135].

In hypermetabolic states such as exercise, sepsis, trauma, and cancer, the release of BCAAs from muscle protein provides a pool of amino acids for the synthesis of priority proteins and/or an important source of oxidative energy [136-138]. Many cancer patients experience involuntary weight loss termed cachexia, which is associated with a loss of skeletal muscle mass [139]. Several *in vivo* tumor models have demonstrated similar cachetic phenotypes. In Walker 256 carcinoma tumor-bearing rat models, an increase in protein degradation and leucine oxidation in skeletal muscle is observed [140,141]. The increase in BCAA oxidation corresponds with an increase in BCKDC activity in the muscle tissue of Walker 256 and Morris hepatoma 5123 tumor-bearing animals [142-144]. Mediators of proteolysis and BCAA oxidation in muscle tissue of cancer patients may involve pro-inflammatory cytokines [142,145]. Shiraki *et al*. assessed the effect of TNF-α administration on BCKDC activity in rat liver [146]. In TNF**-**ɑ**-**treated rats, BCKDC activity was higher than in control due to reduced BDK-mediated phosphorylation and inactivation. Downregulation of BDK mRNA by the glucocorticoid dexamethasone in rat hepatoma cell lines suggests hormonal regulation of BCAA metabolism and BCKDC activity [147]. A recent metabolomics profiling study of men after androgen deprivation therapy for prostate cancer demonstrated a decrease in the products of the BCKDC reaction, further demonstrating a role for hormone control of BCAA metabolism [148]. Interestingly, the thiamine cofactor TPP is a potent inhibitor (Ki = 4 μM) of BDK-mediated phosphorylation, allowing BCKDC to remain active [149]. High-dose thiamine supplementation increases BCKDC activity in thiamine-responsive MSUD patients [135,150]. However, It is unknown what impact thiamine supplementation has on BCKDC activity and BCAA metabolism in cancer patients. Further research is required to establish if any relationship between thiamine supplementation with BCAA metabolism and cancer cachexia exists.

## Conclusions

The alterations in thiamine homeostasis and increase in cancer cell proliferation with thiamine supplementation highlights a significant role for thiamine in cancer. Metabolic studies have provided strong evidence that cancer cells exploit thiamine-dependent enzymes and pathways for anabolic, proliferative, and survival purposes. Unfortunately, how thiamine supplementation impacts the metabolic phenotype of cancer cells is currently hypothetical and is an area of research greatly needed. Refinement of model systems will be absolutely essential in establishing the effects of increasing thiamine supplementation on cancer metabolism and proliferation. Common cell culture media contains super-physiological levels of thiamine that may obscure the importance of thiamine in cancer cell metabolism when using *in vitro* models. For instance, high glucose DMEM contains 10 μM thiamine, which is approximately 1,000 times greater than circulating plasma levels. Additionally, an organismal approach for understanding the role of thiamine in tumor metabolism will need to include the interplay with other nutrients, the tumor microenvironment, and tumor-stromal tissue demands for thiamine. Once connections between thiamine and cancer cell metabolism are established, new opportunities for therapeutic intervention and dietary modification to reduce disease progression in cancer patients will follow.

## Abbreviations

α-KG: α-ketoglutarate; α-KGDH: Alpha-ketoglutarate dehydrogenase; AThTP: Adenosine thiamine triphosphate; BCAA: Branched chain amino acid; BCKDC: Branched chain alpha-keto acid dehydrogenase complex; BDP: α-keto acid dehydrogenase phosphotase; BDK: α-keto acid dehydrogenase kinase; DCA: Dichloroacetate; DMEM: Dulbecco’s modified eagle’s serum; DNC: Deoxynucleotide carrier; DV: Daily value; F6P: Fructose 6-phosphate; 5-FU: 5-fluorouracil; G3P: Glyceraldehyde 3-phosphate; HIF-1α: Hypoxia-inducible factor-1 alpha; hMEC: Human mammary epithelial cell; 1H-NMR: Hydrogen-1-nuclear magnetic resonance; HPLC: High performance liquid chromatography; LDHA: Lactate dehydrogenase A; LLC: Lewis lung carcinoma; LT: Low thiamine; M2-PK: M2 isoform of pyruvate kinase; MSUD: Maple syrup urine disease; NADH: Nicotinamide adenine dinucleotide; NAPDH: Nicotinamide adenine dinucleotide phosphate-oxidase; NF: Normal-fat; NT: Normal thiamine; PDH: Pyruvate dehydrogenase; PDK: Pyruvate dehydrogenase kinase; PPP: Phosphate pathway; RDI: Recommended daily intake; ROS: Reactive oxygen species; R5P: Ribose 5-phosphate; TA: transaldolase; TCA: Tricarboxylic acid; TKT: Transketolase; TKTΔ38: 38 amino acid deletion pseudo-TKTL1; TKTL: TKT-like; TMP: Thiamine monophosphate; TMPase: Thiamine monophosphatase; TNF: Tumor necrosis factor; TPC: Thiamine pyrophosphate carrier; TPK1: Thiamine pyrophosphokinase-1; TPP: Thiamine pyrophosphate; TPPase: Thiamine pyrophosphatase; TTP: Thiamine triphosphate; USDA: United States Department of Agriculture; X5P: Xylulose 5-phsophate.

## Competing interests

All authors declare no financial competing interests.

## Author’s contributions

JZ conceived the review paper, and outline, and was responsible for revising and critically assessing the intellectual content. RS was responsible for critically reviewing the literature and drafting sections and figures within the manuscript. BH was responsible for critically reviewing the literature and drafting sections and figures within the manuscript. SY was responsible for critically reviewing the literature and drafting sections and figures within the manuscript. All authors read and approved the final manuscript.
